# The effect of drinking water pH on the human gut microbiota and glucose regulation: results of a randomized controlled cross-over intervention

**DOI:** 10.1038/s41598-018-34761-5

**Published:** 2018-11-09

**Authors:** Tue H. Hansen, Mette T. Thomassen, Mia L. Madsen, Timo Kern, Emilie G. Bak, Alireza Kashani, Kristine H. Allin, Torben Hansen, Oluf Pedersen

**Affiliations:** 1 The Novo Nordisk Foundation Center for Basic Metabolic Research, Section of Metabolic Genetics, Faculty of Health and Medical Sciences, University of Copenhagen, Copenhagen, Denmark; 2 Department of Cardiology and Endocrinology, Slagelse Hospital, Slagelse, Denmark; 3 Center for Clinical Research and Prevention, Bispebjerg and Frederiksberg Hospital, Copenhagen, Denmark; 4 Faculty of Health Sciences, University of Southern Denmark, Odense, Denmark

## Abstract

Studies in rodent models have shown that alterations in drinking water pH affect both the composition of the gut microbiota and host glucose regulation. To explore a potential impact of electrochemically reduced alkaline (pH ≈ 9) versus neutral (pH ≈ 7) drinking water (2 L/day) on human intestinal microbiota and host glucose metabolism we conducted a randomized, non-blinded, cross-over study (two 2-week intervention periods, separated by a 3-week wash-out) in 29 healthy, non-smoking Danish men, aged 18 to 35 years, with a body mass index between 20.0 to 27.0 kg m-2. Volunteers were ineligible if they had previously had abdominal surgery, had not been weight stabile for at least two months, had received antibiotic treatment within 2 months, or had a habitual consumption of caloric or artificially sweetened beverages in excess of 1 L/week or an average intake of alcohol in excess of 7 units/week. Microbial DNA was extracted from faecal samples collected at four time points, before and after each intervention, and subjected to 16S rRNA gene amplicon sequencing (Illumina MiSeq, V4 region). Glycaemic regulation was evaluated by means of an oral glucose tolerance test.No differential effect of alkaline versus neutral drinking water was observed for the primary outcome, overall gut microbiota diversity as represented by Shannon’s index. Similarly, neither a differential effect on microbiota richness or community structure was observed. Nor did we observe a differential effect on the abundance of individual operational taxonomic units (OTUs) or genera. However, analyses of within period effects revealed a significant (false discovery rate ≤5%) increase in the relative abundance of 9 OTUs assigned to order Clostridiales, family Ruminococcaceae, genus Bacteroides, and species Prevotella copri, indicating a potential effect of quantitative or qualitative changes in habitual drinking habits. An increase in the concentration of plasma glucose at 30 minutes and the incremental area under the curve of plasma glucose from 0 30 and 0 120 minutes, respectively, was observed when comparing the alkaline to the neutral intervention. However, results did not withstand correction for multiplicity. In contrast to what has been reported in rodents, a change in drinking water pH had no impact on the composition of the gut microbiota or glucose regulation in young male adults. The study is registered at www.clinicaltrials.gov (NCT02917616).

## Introduction

The incidence of type 2 diabetes (T2D) is increasing and 592 million people worldwide are projected to be affected by the disease by 2035, which makes T2D a major public-health challenge^1^. The pivotal feature of T2D is hyperglycaemia caused by hepatic- and peripheral insulin resistance, accompanied by progressing beta-cell dysfunction. However, etiologically and pathophysiologically T2D is a heterogeneous disorder with several genetic and environmental factors affecting the risk of developing the diabetic phenotype. Among environmental factors lifestyle-related variables such as physical inactivity and energy-dense dieting have long been recognized as important determinants of T2D development, but other potentially modifiable risk factors have recently been identified, including gut microbiota dysbiosis^2,3^ and dietary acid load^4^.

Experimental lowering of blood pH within the normal range has been shown to reduce insulin sensitivity^5^. A diet rich in acidogenic foods can induce whole-body acid-base imbalance in the form of low-grade metabolic acidosis^6^ and markers of diet-induced acidosis have been associated with insulin resistance^7,8^. Interestingly, in a recent study of 66,485 French middle-aged women dietary acid load predicted incident T2D over a period of 14 years independent of other known risk factors^4^; a result that was independently corroborated in a study of 120′053 Americans^9^.

While early reports of dysbiosis in T2D patients^2,3^ have likely been confounded by pharmaceutical therapy^10^, a recent study of 277 Danes reported specific functional shifts in the faecal microbiome of insulin resistant, non-diabetic individuals^11^. Through integrated analyses of metagenomics and metabolomics, combined with mechanistic studies in mice, *Prevotella copri* and *Bacteroides vulgatus* were identified as bacterial drivers of insulin resistance and aggravated glucose intolerance, thereby substantiating the etiological role of the gut microbiota in T2D pathogenesis. Although it has been demonstrated that dietary nutrient composition is a strong determinant of intestinal ecology^12^, with dietary changes being mirrored by compositional changes within days^13^, it remains elusive whether the microbial features associated with insulin resistance and T2D in humans are induced by specific dietary patterns, including acidogenic diets.

Alkaline drinking water has been proposed by proponents of alternative medicine as a remedy to counteract the effects of an acidogenic diet. While many claims of health promoting effects of alkaline water are scientifically unsubstantiated, changes in drinking water pH have been reported to affect both gut microbiota composition and host metabolism as evidenced by two recent studies in diabetes-prone non-obese diabetic (NOD) mice^14,15^. A study by Wolf and colleagues showed that neutral water (pH ≈ 7) compared to acidic water (pH ≈ 3) increased the incidence of diabetes^14^. Acidic water also decreased the abundance of *Firmicutes* and increased the abundance of *Actinobacteria* and *Proteobacteria*. Contrastingly, a study by Sofi and colleagues found that changing the drinking water from acidic (pH ≈ 3) to neutral (pH ≈ 7) decreased diabetes incidence and rate of disease progression, accompanied by a reduction in the abundance of the genus *Bacteroides* and some species of *Prevotella* (*P. oris*, *P. melaninogenica*, *P. loescheii*, and *P. copri*), whereas other species of *Prevotella* (*P. multiformis* and *P. shahii*) were increased along with species of *Parabacteroides* (*P. goldsteinii* and *P. distasonis*)^15^. Differences in baseline gut microbiota, along with difference in other environmental circumstances, have been proposed as an explanation for the conflicting results regarding diabetes incidence and rate of progression^16,17^. Importantly, both studies showed substantial differences in the gut microbiota of mice drinking acidic and neutral water, demonstrating that drinking water pH can have a profound impact on the gut microbial community. NOD mice are the most commonly used rodent model of autoimmune diabetes^18^. Interestingly, a recent study has shown that NOD mice develop insulin resistance, with hepatic insulin resistance being present already in the pre-diabetic phase and muscle insulin sensitivity being impaired as early as three days after onset of diabetes^19^. Taken together these observations suggest that the effects of changes in drinking water pH reported by Wolf *et al*. and Sofi *et al*. may in part be influenced by mechanisms affecting insulin resistance in addition to augmented autoimmune insulitis.

In the present study, we investigated possible compositional changes in the gut microbial community and glucose regulation of young healthy adults following intake of alkaline vs. neutral drinking water.

## Methods

### Subjects and study design

Volunteers were recruited by advertisement via online resources and by poster-advertisement at the University of Copenhagen campuses. Healthy, non-smoking men, aged 18 to 35 years, with a body mass index between 20.0 to 27.0 kg·m^−2^ were eligible for inclusion. Volunteers were ineligible if they had previously had abdominal surgery, had not been weight stabile for at least two months, had received antibiotic treatment within 2 months, or had a habitual consumption of caloric or artificially sweetened beverages in excess of 1 L/week or an average intake of alcohol in excess of 7 units/week. Participants in need of medical treatment during the study period were excluded.

**Figure 1:**
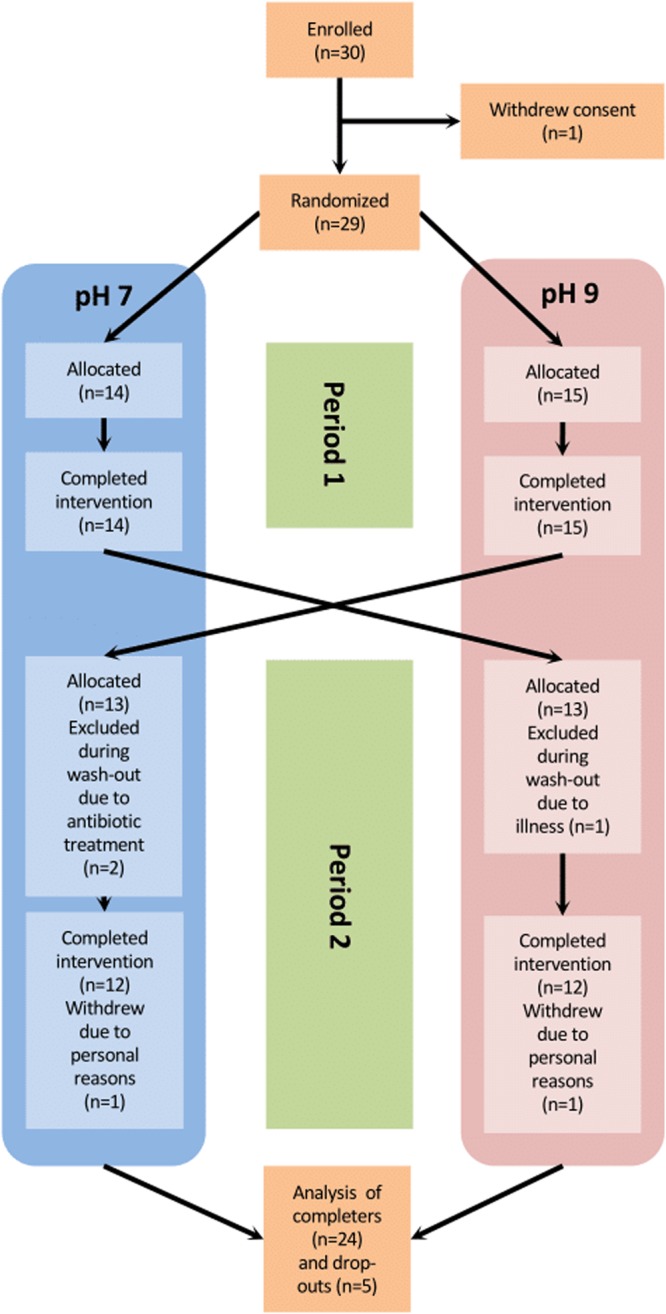
Flowchart.

A total of 30 volunteers were included in a non-blinded, randomized cross-over study with two 2-week intervention periods with an interposed washout period of at least 3 weeks. Participants were examined before and after each intervention period (Fig. 1). Treatment sequence was assigned at random by use of a computer algorithm. Generation of allocation sequence, enrolment of participants and assignment to interventions was conducted by THH and MTT. During intervention periods participants were instructed to quench thirst by drinking the experimental water. Participants were required to ingest a minimum of two litres distributed throughout the day, and record the intake on a daily basis. Intake of regular tap water during the intervention periods was prohibited. Intake of warm water-based beverage (e.g. tea and coffee) was allowed, but had to be based on the experimental water to the greatest extent possible. A limited amount of alcohol (not exceeding 7 drinks/week) was accepted. Participants were provided with a portable, air-tight container for the purpose of quenching thirst outside the home. In the 3-week washout period between the two intervention periods participants were instructed to return to their normal drinking habits. Compliance during intervention periods was ensured by regular e-mail and text message reminders.

The study was approved by the Regional Committee on Health Research Ethics for the Capital Region of Denmark (Protocol# H-15011825) and conducted according to the Helsinki Declaration. Written informed consent was obtained from all participants. The study was registered retrospectively at clinicaltrials.gov (NCT02917616; https://clinicaltrials.gov/ct2/show/NCT02917616) on September 26, 2016.

### Production of experimental water

Each participant was provided with a validated commercial device (PUREPRO USA CORP, IL USA) capable of producing water with specific pH by means of ion exchange and electrolysis, to be used during each intervention period. The devices were installed on the tap in the participants’ homes, which were all located in the Copenhagen area. For each participant, the same device was used during both intervention periods. During each installation, the device was tested and duplicate pH measures were made on the setting used for the intervention. For the intervention with neutral drinking water the mean pH was 7.19 (range 7.01–7.38) and for the intervention with alkaline drinking water mean pH was 9.06 (range 8.64–9.44), corresponding to a mean difference in pH of 1.87 (range 1.55–2.33) between interventions.

### Examinations

Participants were examined in the morning following a 10-hour overnight fast, before and after each intervention period. Examinations took place at the Novo Nordisk Foundation Center for Basic Metabolic Research, Unit for Physiological Studies located at Frederiksberg Hospital, Frederiksberg, Denmark.

#### Gastrointestinal symptoms

Gastrointestinal symptoms were assessed by a digital symptom questionnaire using a visual analogue scale, containing registration of overall gastrointestinal symptoms, bloating, abdominal pain, constipation, diarrhoea, flatulence, metallic taste, stool consistency, nausea, tiredness and bowel habits.

#### Anthropometrics

Participants were weighed on an electronic scale (TANITA BC-420MA, Tanita Corporation of America, IL USA) without shoes, dressed in light clothing or underwear. Height was measured to the nearest 0.5 cm without shoes using a wall-mounted stadiometer (ADE MZ10023, ADE, Germany). Waist and hip circumference was measured to the nearest cm in erect position midway between the iliac crest and the lower costal margin and at the level of the pubic symphysis, respectively. Sagittal abdominal diameter was measured in the supine position at the high point of the iliac crest during exhalation. BMI was calculated by dividing the weight (kg) by the square of the height (m) and body fat percentage was assessed with bioelectric impedance analysis (TANITA BC-420MA). Blood pressure was assessed in the supine position following a 5-minute rest as the average of 3 repeated measurements made by an automatic sphygmomanometer (A&D Medical, Japan).

#### Urine

Second void urine samples were collected in the fasting state and pH was measured immediately after collection using a calibrated pH meter (HI 99192, Hanna Instruments, RI USA).

#### Blood

Venous blood was collected in the fasting state and analysed for concentrations of plasma glucose, serum insulin, and plasma high-sensitivity C-reactive protein (hsCRP). Plasma glucose was analysed using the hexokinase method (GLUC3, Roche/Hitachi Cobas e501), serum insulin was analysed using an enzyme-linked chemiluminescent immunoassay (Insulin, Roche/Hitachi Cobas e401), and hsCRP was analysed using a particle-enhanced turbidimetric immunoassay (CRPHS, Roche/Hitachi Cobas e701). Following fasting blood sampling the participants were asked to consume 75 g of glucose dissolved in 250 mL of cold water over 5 minutes. Blood was sampled at 0, 30, 60, 90 and 120 minutes. Blood samples obtained during the OGTT were analysed for plasma glucose and serum insulin, as described.

#### Faeces

Faecal samples were collected before and after each intervention following standardized operating procedures, including home sampling with immediate freezing at −18 °C and transfer on dry ice for final storage at −80 °C within 48 hours.

### DNA extraction, 16S rRNA library preparation and sequencing

Genomic DNA was isolated from 200 mg of faeces using the NucleoSpinSoil kit (Macherey-Nagel GmbH & Co. KG, Germany) following the manufacturer’s instruction. For the cell lysis buffer SL2 + Enhancer buffer SX were used, the subsequent vortex step was replaced with repeated bead beating. DNA yield, purity and integrity were assessed using a Qubit 2.0 fluorometer, a NanoDrop 2000 spectrometer (Thermo Fisher Scientific Inc., MA USA) and agarose gel electrophoresis. Library preparation with polymerase chain reaction (PCR) amplification was performed using 20 ng bacterial DNA, 0.2 μM of each barcoded forward and reverse primer, and HotMasterMix (5Prime) solution in a total volume of 25 μL. To target the variable region 4 (V4) of the 16S rRNA gene a forward primer 515 F (5′ AATGATACGGCGACCACCGAGATCTACAC <i5> TATGGTAATTGTGTGCCAGCMGCCGCGGTAA 3′) and a reverse primer 806 R (5′AAGCAGAAGACGGCATACGAGAT <i7> AGTCAGTCAGCCGGACTACHVGGGTWTCTAAT 3′) were used; each primer consists of the appropriate Illumina adapter, an 8-nucleotide index sequence i5 and i7, a 10-nucleotide pad sequence, a 2-nucleotide linker, and the gene-specific primer^20,21^. The PCR reaction was performed in a Thermocycler (Eppendorf AG, Germany), using the following parameters: 3 minutes at 94 °C, followed by 28 cycles of 20 seconds at 94 °C, 30 seconds at 55 °C and 54 seconds at 72 °C. The samples were purified with a magnetic-bead based clean-up and size selection kit (Macherey-Nagel GmbH & Co. KG, Germany). Amplicons were visualized by gel electrophoresis and quantified by a Qubit 2.0 fluorometer. A master DNA pool was generated from the purified products in equimolar ratios. The DNA was sequenced using an Illumina MiSeq platform (MiSeq Reagent Kits v2, 500 cycles), generating a total of 10,862,648 (range 33,320–424,278) paired-end reads, which were merged using FLASH^22^, generating contigs comprising 250 ± 6 base pairs. Expected error filtering (Emax = 0.5) in USEARCH^23^ was used to exclude low quality contigs. Using Quantitative Insights Into Microbial Ecology (QIIME) v.1.5^20^ the remaining high-quality contigs (3,644,107; range 10,906-153,524) were de-multiplexed and assigned to operational taxonomic units (OTU) by a minimum 97% sequence similarity against Greengenes v.13.8^24^ using closed reference OTU picking.

### Statistical analyses

All statistical tests were performed using R v3.3.1 (www.r-project.org). No *a priori* sample size calculation was made. The primary outcome was difference in Shannon diversity of species level OTUs after the alkaline intervention relative to the neutral water intervention. Secondary outcomes included between- and within-intervention changes in richness, community structure and membership, and taxonomic composition. Whereas nominal P-values ≤0.05 are reported, analyses were adjusted for multiplicity *ad modum* Benjamini-Hochberg^25^ and a false discovery rate (FDR) ≤5% was observed for significance.

### *Intervention effect on p*hysiological outcomes

Between-intervention effect (pH9 vs. pH7) on physiological outcomes was analysed by analysis of covariance (ANCOVA) in a linear mixed model framework with subject as a random factor. Models were fitted using restricted maximum likelihood and include a treatment × visit interaction with a level for each combination of treatment (pH7 or pH9) and visit (1 through 4), allowing a) participants to have individual baselines in the first intervention period and b) for the baseline in the second period to depend on the preceding treatment, thereby taking into account inadequate randomization and potential carry-over effects, respectively. Treatment effect was estimated by a *post-hoc* t-test as the average difference between pre- and post-intervention visits within a period. For outcomes measured repeatedly at each visit (i.e. glucose and insulin during OGTT) a three-way time × treatment × visit interaction was included, instead of the two-way treatment × visit interaction, and a Gaussian spatial correlation structure was applied, assuming repeated measurements to be serially correlated within a visit. The global treatment effect on plasma glucose and serum insulin during an OGTT was assessed using a χ^2^ test. Model assumptions were checked visually by inspection of residual plots (homoscedasticity) and normal probability plots (Gaussian distribution). Logarithmic transformation of the dependent variable was applied where appropriate. Areas under the curve (AUC) were calculated using the trapezoidal method and incremental values represent the expression above fasting values. Indices of beta-cell function and insulin resistance were calculated as described in Table S1. Difference in compliance between intervention periods among study completers was tested using a Wilcoxon signed-rank test.

### Intervention effect on gut microbiota composition and diversity

Downstream analyses of 16S sequencing data were performed in R using the *phyloseq* package v1.16.2^26^. Prior to alpha- and beta-diversity analyses samples were rarefied to an equal sequencing depth of 10,306 reads. For analysis of between-intervention effect at OTU level, a set of core OTUs with a mean relative abundance ≥0.05% and present in at least one sample in ≥50% of participants were selected. Similarly, genus level analyses included all genera present at least once in ≥50% of participants. Between-intervention effect on richness, alpha-diversity indices and relative abundance of individual OTUs and genera was tested using linear mixed models (as for physiological outcomes), following logarithmic transformation. When testing the treatment effect on a given taxa, a pseudo count equal to the lowest detected relative abundance of that taxa was added prior to logarithmic transformation. Participants in which a given taxon was not present in any of the samples were excluded from the analyses of that taxon in order to minimize the effect of zero-inflation. Between-intervention effect on community structure was assessed by principal coordinate (PCoA) ordination using Bray-Curtis, unweighted- and weighted UniFrac metrics, and analysed using permutational multivariate analysis of variance (PERMANOVA) as implemented in the vegan R package v2.4.0. Analyses of within-intervention effect was analysed in a mixed linear model framework contrasting pre- and post-intervention measurements with a random effect of subject and adjusted for intervention sequence. For analyses considering interventions as technical replicates (as if participants received the same intervention twice) we applied a mixed linear model contrasting pre- and post-intervention measurements adjusted for intervention sequence, with a random effect of subject within period.

## Results

The study was carried out from November 2015 until April 2016. A total of 30 male participants were enrolled; however, one participant withdrew consent prior to randomization. Twenty-nine participants completed the first intervention period, and 24 completed both periods; 3 were excluded due to intercurrent illness, and 2 withdrew from the study due to personal circumstances (Fig. 1). Data from all available time points are included in the analyses.

### Gut microbiota

We did not observe an effect of changing drinking water pH on overall diversity as represented by Shannon’s index which was the primary outcome. Similarly, no effect was observed for OTU based richness, estimated richness (Chao1) or Simpson’s reciprocal index (Fig. S1) when comparing the alkaline and neutral water interventions. However, in *post hoc* analyses comparing pre- and post-intervention samples for each intervention we observed a 15.8% (95% CI: 6.9–25.5%; P = 0.0003) increase in observed richness and a 15.4% (95% CI: 5.3–26.5%; P = 0.002) increase in estimated (Chao1) richness after the neutral-water intervention which remained significant after correction for multiple testing (Q = 0.003 and 0.008, respectively). Likewise, there was a 9.5% (95% CI: 0.25–19.6%; P = 0.04) increase in overall diversity represented by the Shannon index, although the effect was not statistically significant after correction for multiplicity. We did not observe any change in richness or overall diversity during the alkaline-water intervention.

**Figure 2:**
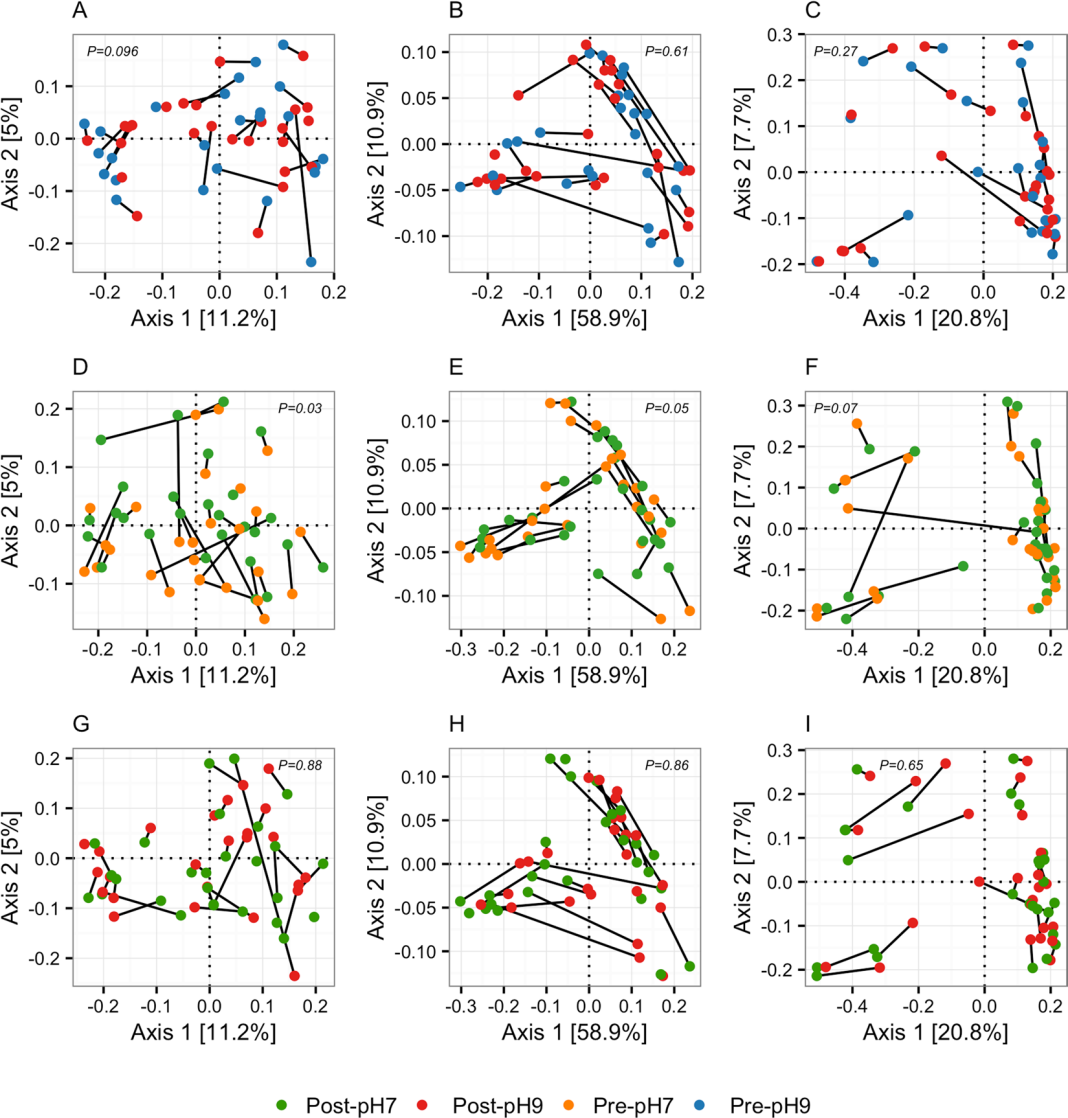
Principal coordinate plots of faecal microbiota community structure. Principal coordinate plots (axes 1 and 2) of treatment effect on community structure based on unweighted UniFrac (**A,D,G**), weighted UniFrac (**B,E,H**), and Bray-Curtis distances (**C,F,I**). Samples from the alkaline (**A–C**) and neutral water intervention (**D–F**), as well as post-intervention samples from both periods (**G**–**I**) are depicted separately. Samples from the same participant are connected by solid lines. P-values are from permutational multivariate analysis of variance.

Principal coordinate ordination of community structure and community membership as assessed by Bray-Curtis dissimilarity and UniFrac (unweighted and weighted) distances revealed highly individual responses to both the alkaline and neutral water interventions (Fig. 2). The effect of alkaline water relative to neutral water on community structure was analysed by comparing post-intervention samples from the two periods (Fig. 2G–I). Using PERMANOVA analysis of Bray-Curtis dissimilarity and UniFrac distances, we did not observe any difference in effect between the two interventions. Within the individual intervention periods there was an effect (P = 0.03) of the neutral water on community membership as assessed by unweighted UniFrac (Fig. 2D), but it did not remain statistically significant when adjusted for multiplicity (Q = 0.21). We did not observe any effect on community structure or community membership during the alkaline intervention (Fig. 2A–C).

**Figure 3:**
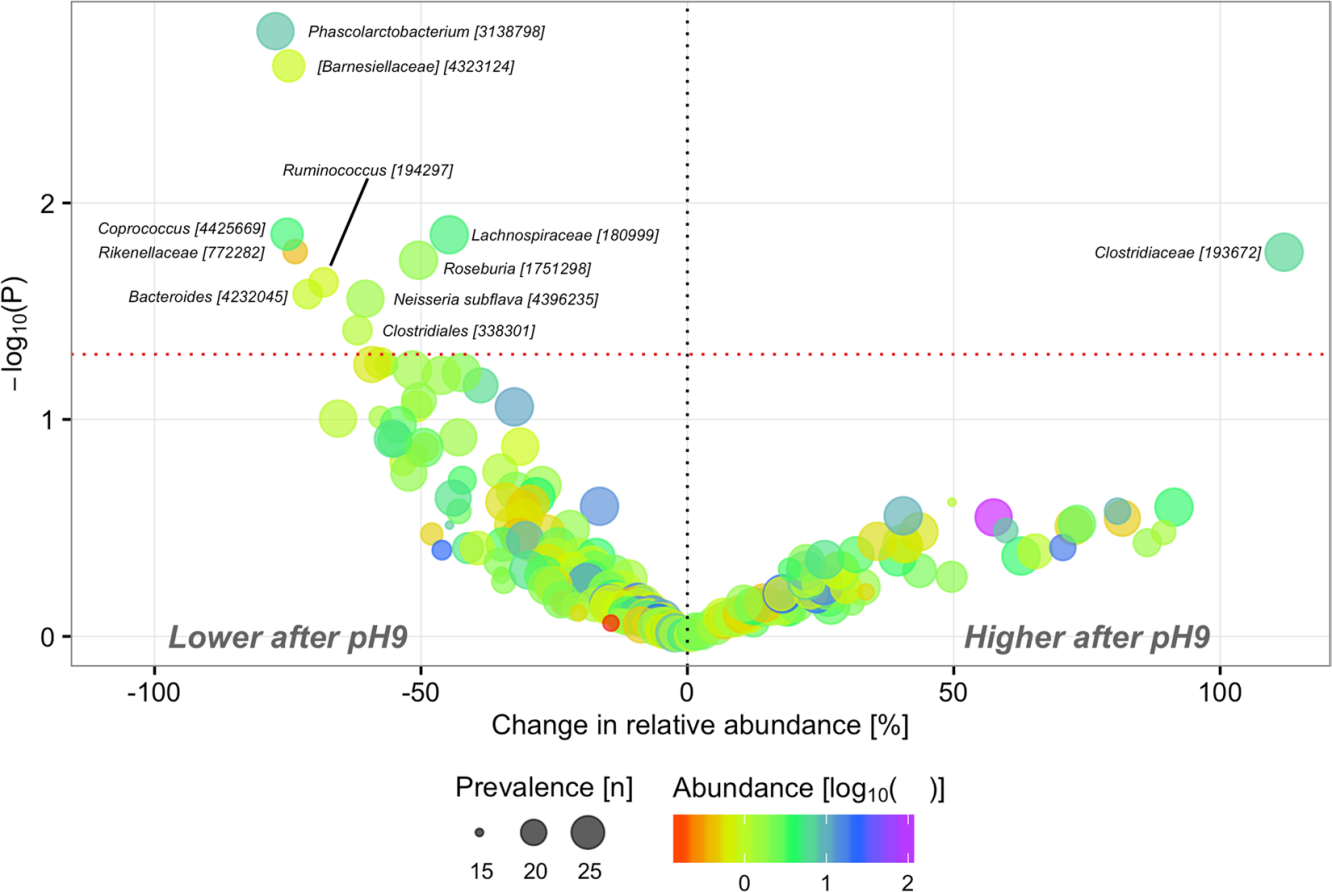
Treatment effect on OTU composition. Change (%) in geometric means of relative abundance and corresponding P-values derived from linear mixed models of treatment effect i.e. alkaline versus neutral water. Prevalence indicates number of participants in which a given OTU is present in at least one sample. Abundance indicates mean relative abundance of a given OTU in baseline samples. Taxonomy of OTUs [Greengenes ID] is given at the lowest classified rank. Only OTUs with P ≤ 0.05 are annotated.

Baseline composition of the core microbiota (Figs S2 and S3) was dominated by members of the two major phyla *Bacteroidetes* (49.7%) and *Firmicutes* (45.4%) with *Prevotellaceae* (24.9%), *Lachnospiraceae* (20.7%), *Bacteroidaceae* (18.6%) and *Ruminococcaceae* (15.6%) as the predominant families, albeit with substantial inter-individual variation. We did not observe any significant (FDR ≤5%) differential effect of the two interventions at OTU level; however, at nominal significance 10 OTUs were less abundant and one OTU was more abundant after the alkaline intervention than after the neutral intervention (Fig. 3). Aggregated at genus level results were similar with no FDR significant effects of alkaline- vs. neutral water (Table S2).

**Figure 4:**
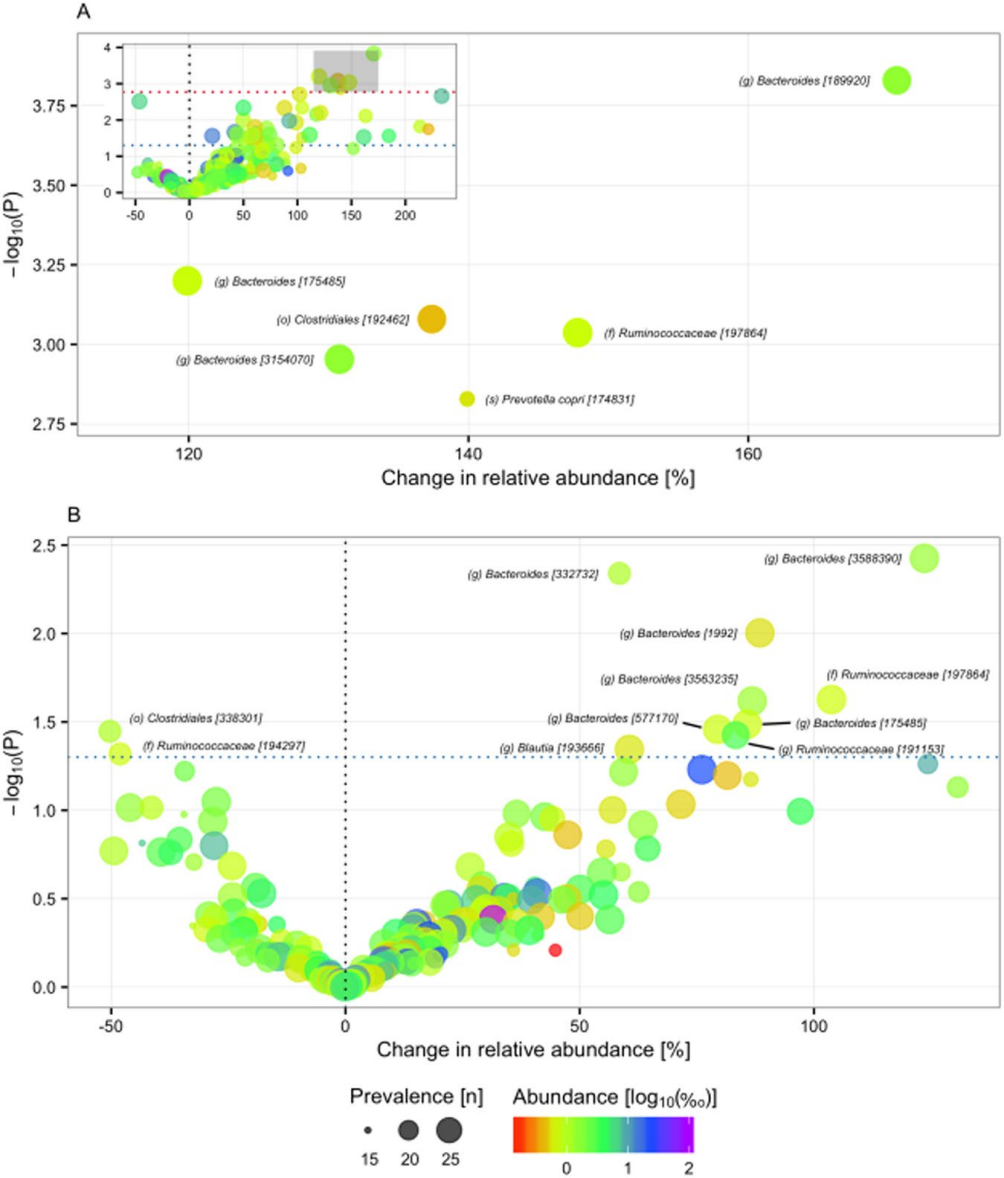
Intervention effect on OTU composition. Change in relative abundance (% increase/decrease in geometric mean) of core OTUs during the neutral water (**A**) and alkaline (**B**) water interventions. Red dotted line indicates the cut-off corresponding to a Q-value of 0.05, and the blue dotted line indicates a P-values of 0.05. Prevalence indicates number of participants in which a given OTU is present in at least one sample. Abundance indicates mean relative abundance (‰) of a given OTU in baseline samples. Taxonomy of OTUs [Greengenes ID] is given at the lowest classified rank. o, order. f, family. g, genus.

Looking at change in relative abundance of individual OTUs within each intervention period, we observed an effect of neutral water (P ≤ 0.05) on 40 out of 201 core OTUs, six of which were significant at the pre-specified FDR of 5% (Fig. 4A): three assigned to genus *Bacteroides*, one assigned to species *Prevotella copri*, one assigned to family *Ruminococcaceae*, and one assigned to order *Clostridiales*. We also observed an effect of the alkaline water intervention on the relative abundance of 11 core OTUs; but none were significant when adjusted for multiplicity (Fig. 4B). Interestingly, the increase in relative abundance of three OTUs assigned to genus *Bacteroides*, one assigned to genus *Blautia* and one to family *Ruminococcaceae* reached nominal significance during both periods, indicating a potential common effect of the two interventions (Fig. S4). Furthermore, when considering paired samples from both interventions as technical replicates, we observed a significant increase (FDR <5%) in relative abundance of nine OTUs (Fig. S5) assigned to order *Clostridiales* (2), family *Ruminococcaceae* (1), genus *Bacteroides* (5) and species *Prevotella copri* (1). We did not observe any genera of which the relative abundance changed significantly within either intervention period (Table S3).

### Glucose regulation, low-grade inflammation and urine pH

**Figure 5:**
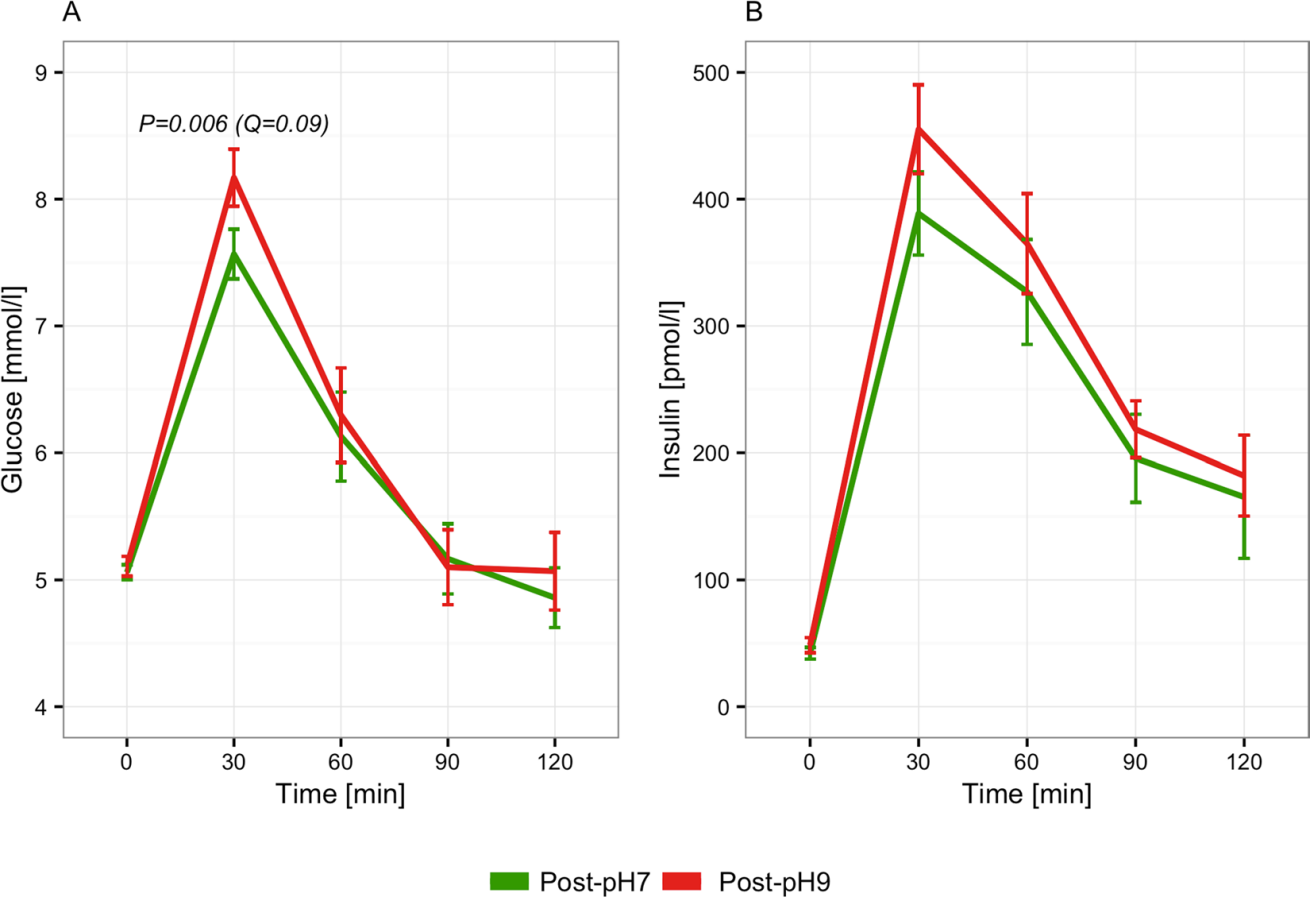
Treatment effect on glucose and insulin during an oral glucose tolerance test. Curves are mean (±SEM) plasma glucose (**A**) and serum insulin (**B**) sampled at 5 time-points during an oral glucose tolerance test. P-value for difference in plasma glucose at 30 min from linear mixed model adjusted for age and body-mass index.

All participants were glucose tolerant and had normal fasting glucose at the baseline examination (Table 1). A global test, considering all sampled time-points during an OGTT, did not show a significant between-intervention difference in neither plasma glucose (P = 0.13) nor serum insulin (P = 0.53) concentration after the two interventions (Fig. S6A,B). Similarly, when considering stimulated glucose and insulin only (i.e. 30 through 120 minutes) there was no significant effect of alkaline water compared to neutral (P = 0.07 and 0.39 for glucose and insulin, respectively). Considering individual time-points separately, our analyses showed that plasma glucose was 9.3% (95CI: 16.5–2.6%) higher at 30 minutes after the alkaline water intervention compared to the neutral water intervention (Fig. 5). Correspondingly, incremental AUC of glucose from 0 to 30 minutes, and from 0 to 120 minutes, were higher (9.3% (16.5–2.6%; P = 0.006) and 41.9% (97.2–2.2%; P = 0.04), respectively), whereas AUCs unadjusted for fasting glucose were not. Importantly, none of the observed effects on plasma glucose withstood correction for multiplicity at a predefined FDR of 5%. There was no detectable effect of alkaline compared to neutral water on serum insulin concentration, nor did we observe an effect on indices of beta-cell function or insulin resistance (Table 2). Similarly, there was no discernible effect of alkaline compared to neutral water on hsCRP (P = 0.89). There was no measurable effect of alkaline water compared to neutral on fasting urine pH (−0.9% (95CI: –5.1–3.5%; P = 0.69)).

**Table 1:** Baseline Characteristics.

	Randomized (n = 29)
**Demographics**	
Age (y)	23.0 (21.0–26.0)
**Anthropometrics**	
Body-mass index (kg∙m^−2^)	22.7 (21.2–24.6)
Body fat (%)	15.9 (12.10–18.0)
Sagittal abdominal diameter (cm)	18.0 (17.5–19.5)
**Blood pressure**	
Systolic (mmHg)	122 (118–130)
Diastolic (mmHg)	68 (62–72)
**Glucose regulation**	
Fasting glucose (mmol/L)	5.2 (5.0–5.4)
2-h glucose (mmol/L)	4.5 (4.3–5.4)
Impaired fasting glycaemia (n)	0
Impaired glucose tolerance (n)	0
**Lifestyle**	
Current smokers (%)	0
Physical activity (min∙week^−1^)	755 (525–945)

Data is presented as median (interquartile range) or number of participants within a category.

**Table 2:** Effect of alkaline versus neutral water on glycaemic variables.

	Effect (%)	95% CI	P	Q
**Plasma glucose**				
Glucose_0_	−1.4	−5.7–3.1	0.5	0.74
Glucose_30_	9.3	2.6–16.5	0.006	0.09
Glucose_60_	11.0	−1.0–24.5	0.08	0.35
Glucose_90_	0.0	−9.2–10.1	0.99	0.99
Glucose_120_	2.3	−10.1–16.5	0.73	0.81
AUC_Glucose_	4.9	−1.1–11.3	0.11	0.56
AUC_Glucose(0–30)_	3.1	−1.2–7.5	0.16	0.59
IAUC_Glucose_	41.9	2.2–97.2	0.04	0.46
IAUC_Glucose(0–30)_	9.3	2.6–16.5	0.006	0.15
**Serum insulin**				
Insulin_0_	−4.1	−26.2–24.6	0.75	0.81
Insulin_30_	14.1	−6.3–38.8	0.19	0.44
Insulin_60_	25.9	−6.4–69.3	0.13	0.36
Insulin_90_	6.5	−19.6–41.0	0.66	0.81
Insulin_120_	15.1	−23.2–72.4	0.50	0.74
AUC_Insulin_	13.7	−5.3–36.4	0.17	0.59
AUC_Insulin(0–30)_	10.1	−8.8–33.0	0.32	0.61
IAUC_Insulin_	19.1	−2.0–44.8	0.08	0.56
IAUC_Insulin(0–30)_	14.1	−6.3–38.8	0.19	0.59
**Indices of insulin secretion and β−cell function**				
AUC_Insulin_/AUC_Glucose_	8.2	−7.1–26.1	0.31	0.61
IAUC_Insulin_/IAUC_Glucose_	−16.4	−40.6–17.8	0.31	0.61
AUC_Insulin(0–30)_/AUC_Glucose(0–30)_	6.8	−10.3–27.2	0.46	0.82
IAUC_Insulin(0–30)_/IAUC_Glucose(0–30)_	4.3	−13.4–25.6	0.66	0.92
HOMA2-B	0.0	−13.3–15.4	0.99	0.99
IGI	−16.3	−37.5–12.1	0.23	0.61
CIR	−12.7	−30.6–10.0	0.25	0.61
DI	−20.8	−40.0–4.5	0.10	0.56
1^st^ phase insulin release	−3.5	−17.2–12.4	0.65	0.92
2^nd^ phase insulin release	−0.8	−12.7–12.7	0.90	0.98
BIGTT-AIR_30_	2.2	−12.1–18.8	0.78	0.97
**Indices of insulin sensitivity**				
HOMA2-IR	−4.7	−27.0–24.6	0.73	0.96
ISI_Matsuda_	−6.4	−25.1–17.0	0.56	0.88
MCR	−0.6	−9.4–9.1	0.90	0.98
Stumvoll	−0.4	−10.4–10.8	0.95	0.99
QUICKI	1.7	−3.9–7.7	0.56	0.88
OGIS	−0.4	−5.6–5.2	0.90	0.98

Effect estimates are differences (%) in geometric means (alkaline relative to neutral water) derived from linear mixed models adjusted for age and BMI (except the BIGTT-AIR30 and OGIS indices which were adjusted for age only). A description of the indices is available in Table S1. AUC, area under the curve. IAUC, incremental area under the curve. DI, disposition index. IGI, insulinogenic index. CIR, corrected insulin release. HOMA2-B, homeostatic model assessment of beta-cell function. HOMA2-IR, homeostatic model assessment of insulin resistance. MCR, metabolic clearance rate. QUICKI, quantitative insulin sensitivity check index. OGIS, oral glucose insulin index. ISI, Insulin sensitivity index.

### Gastro-intestinal symptoms

We did not observe any difference in frequency of bowel movements, nor in severity of gastro-intestinal symptoms when comparing alkaline and neutral water interventions (Fig. S7A,B). However, when comparing pre- and post-intervention records, disregarding the type of intervention, VAS scores of abdominal discomfort and degree of constipation were increased during intervention by 71.6% (95CI: 10.4–166.6%; P = 0.02) and 42.0% (95CI: 3.5–94.8%; P = 0.03), respectively (Fig. S7C); albeit the effects did not withstand correction for multiplicity of testing (Q = 0.09 in both cases).

### Compliance

Daily records of water consumption demonstrated that participants on average met the required intake of 2 L per day on 12 out of 14 days (range 8–14 days) during each intervention period. There was no indication of difference in compliance during the two interventions, among those who completed both intervention periods (P = 0.57, Wilcoxon signed-rank test).

## Discussion

In the present study, the objective was to investigate whether changes in drinking water pH has a short-term impact on the intestinal microbial community, as well as glucose regulation of the host. During two periods of two weeks each, participant drank a minimum of 2 L of either alkaline (pH9) or neutral (pH7) water daily. When comparing the two interventions we did not observe any significant effect on neither gut microbiota, glucose regulation, nor low-grade inflammation.

In the present study we included healthy male volunteers in an attempt to reduce potential confounding effects related to sex, demographics and health status. Consequently, we cannot rule out that a change in drinking water pH might affect the composition of the gut microbiota in the case of pre-existing dysbiosis. Furthermore, it is possible that an effect on glucose metabolism might be more pronounced in insulin resistant individuals or individuals with reduced beta-cell function.

The composition of the gut microbiota at baseline was comparable to what has previously been reported in healthy individuals, both in terms of average abundances of the major taxa, as well as inter-individual variation^27^. As pointed out by Wolf *et al*.^14^ and Sofi *et al*.^15^ the contrasting results observed in NOD mice are possibly ascribable to baseline variation in the gut microbiota between facilities. Similar limitations apply when studying free-living humans, in which inter-individual compositional variation at baseline and intra-individual temporal variation is presumably much larger than in laboratory mice.

A recent study by Murakami and colleagues investigated the effect of bicarbonate rich mineral water vs. tap water on glucose regulation and gut microbiota composition in 19 healthy volunteers (12 women)^28^. They reported a significant decrease in glycoalbumin, as well as an increase in the relative abundance of the families *Christensenellaecae*, *Dehalobacteriaceae*, *Bacteroidaceae*, *Porphyromonadaceae*, *Rikenellaceae*, *Erysipelotrichaceae*, *Oxalobacteraceae* and a decrease in *Bifidobacteriaceae* following consumption of 500 ml of bicarbonate rich water for 1 week. Of notice, the concentration of bicarbonate was 100-fold higher in the bicarbonate rich water compared to tap water, yet pH was lower (7.07 compared to 7.58 in tap water). This highlights important aspects regarding the potential effects of variation in drinking water pH. The pH as a measure of hydrogen ion activity of an aqueous solution says nothing about the buffering capacity of said solution. For instance, a solution of sodium bicarbonate is many times more efficient in buffering the acidic environment in the stomach than a solution of sodium hydroxide with the same pH. Given recent results showing an increase in families *Streptococcaceae* and *Micrococcacaea* associated with the use of proton pump inhibitors^29,30^, it is reasonable to assume that the potential effect of drinking water on the gut microbiota is linked to the buffering capacity. Mineral composition of the water may also have an effect on gut microbiota composition, irrespective of the acid-base properties. Divalent cations affect the stability of the outer membrane of Gram-negative bacteria^31^ and are involved in motility, transport, and cell differentiation processes^32^. In the present study, we used an electrochemical process to produce water with neutral and alkaline pH. Due to financial limitations, the mineral content of the experimental water consumed by individual participants was not assessed systematically.

As has recently been demonstrated using population-based datasets^33^, intervention studies aiming to identify a microbiome shift specific to a known association are likely to require considerable sample sizes. This is a first-in-man study and due to the unavailability of relevant effect size estimates no *a priori* sample size calculation could be made when designing the study. Thus, our findings are exploratory and it cannot be ruled out that a larger sample size would have shown a significant difference between the two interventions. For instance, *post hoc* analyses indicated that the present study had 19.5% statistical power to detect (paired t-test) a significant decrease in alpha diversity (Shannon’s index) at a two-tailed α = 0.05 given the observed effect size^34^. Consequently, a study designed to test the specific hypothesis of an effect of alkaline vs. neutral drinking water on alpha diversity would require a sample size of 180 individuals to have 80% statistical power at an α = 0.05.

Limitations of the present study include certain aspects of its design. Participants were instructed to maintain their usual diet pattern. Still, any unintended changes in diet during the study period could potentially influence both the gut microbiota and glucose regulation, obscuring any actual effect of changing drinking water pH. Participants and researchers were non-blinded to intervention sequence which could potentially result in biased estimates of treatment effects, especially of self-reported, subjective outcomes like gastro-intestinal symptom severity. We sought to ensure compliance by regular text message reminders and self-reporting of consumed water amounts, but no objective assessment of adherence to the interventions was made.

In *post hoc* analyses of changes in microbiota composition within each intervention arm we observed a significant increase in the relative abundance of six OTUs, as well as a significant increase in observed and estimated richness, during the neutral water intervention. No significant effects were observed during the alkaline interventions. Of the OTUs that were significant during the neutral water intervention five were among the OTUs with P-values below the 10^th^ percentile during the alkaline intervention, two of which were nominally significant. Consequently, when analysing samples from the two interventions as technical replicates we observed nine OTUs that became more abundant when changing from regular tap water to experimental water. A possible explanation for this apparent effect could be that participants drank more during intervention periods than they usually did, because a minimum daily intake of 2 litres was required. Another possible explanation is that regular beverages were substituted by water during intervention periods. Seventy six percent of participants in the present study were habitual coffee drinkers. The effects of coffee (decaffeinated as well as caffeinated) on gastro-intestinal physiology include increased gastric acid secretion, gall-bladder contraction and rectosigmoid motility^35^. Parallel to these stimulatory effects coffee consumption has been shown to alter the gut microbiota composition in high-fat-fed rats^36^ and increase the abundance of *Bifidobacterium spp*. in humans^37^. Change in coffee consumption during interventions was not recorded and any effect of beverage substitution is speculative only. It is also possible that the observed compositional change is caused by a machine effect irrespective of the chosen pH; however, the exact nature of such an effect is unknown.

## Conclusions

The present study did not show any differential effect of alkaline vs. neutral drinking water on the gut microbiota composition or glucose regulation and inflammatory state of healthy, male volunteers. However, larger studies are required in order to definitively rule out any effect of variation in drinking water pH on the gut microbiota or physiology of the host. Analyses of within intervention changes in microbiota composition indicated a possible effect of quantitative or qualitative changes in habitual drinking habits which requires further experimental elucidation.

### Ethics approval and informed consent

The study was approved by the Regional Committee on Health Research Ethics for the Capital Region of Denmark (Protocol# H-15011825) and conducted according to the Helsinki Declaration. Written informed consent was obtained from all participants. The study was registered at www.clinicaltrials.gov (NCT02917616) on September 28, 2016.

## Electronic supplementary material

Supplementary material

## Data Availability

The datasets are available from the corresponding author on reasonable request.
