# Supplementary material for: The effect of drinking water pH on the human gut microbiota and glucose regulation: results of a randomized controlled cross-over intervention
Source: Sci Rep. 2018 Nov 9;8:16626. doi: 10.1038/s41598-018-34761-5 (PMC6226457; doi:10.1038/s41598-018-34761-5)
Supplement: Supplementary file 1 — Supplementary material [file 41598_2018_34761_MOESM1_ESM.pdf]

SUPPLEMENTARY TABLES AND FIGURES

THE EFFECT OF DRINKING WATER PH ON THE HUMAN GUT MICROBIOTA AND GLUCOSE REGULATION: RESULTS OF A RANDOMIZED CONTROLLED CROSS-OVER INTERVENTION

Tue H. Hansen[1], Mette T. Thomassen[1], Mia L. Madsen[1], Timo Kern[1], Emilie G. Bak[1], Alireza Kashani[1], Kristine H. Allin[1,2], Torben Hansen[1,3], Oluf Pedersen[1]

[1] The Novo Nordisk Foundation Center for Basic Metabolic Research, Section of Metabolic Genetics, Faculty of Health and Medical Sciences University of Copenhagen, Denmark

[2] Department of Clinical Epidemiology, Bispebjerg and Frederiksberg Hospital, the Capital Region, Copenhagen, Denmark

[3] Faculty of Health Sciences, University of Southern Denmark, Odense, Denmark

FIGURE S1. FOREST PLOT OF INTERVENTION EFFECT ON ALPHA DIVERSITY

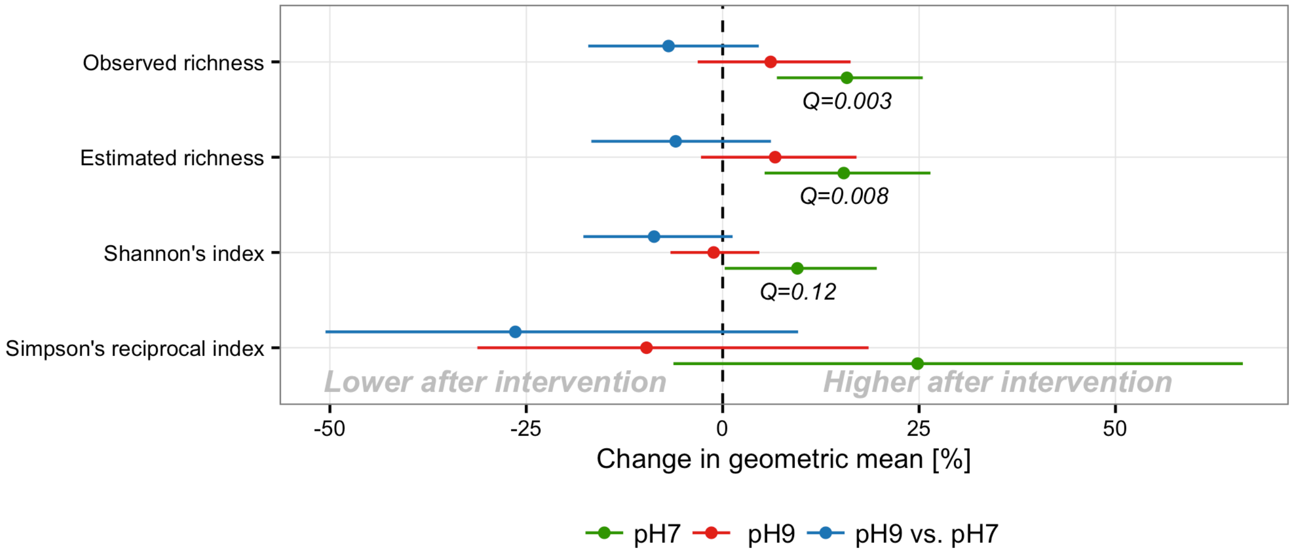

Forest plot of effect estimates (% change in geometric means) and 95% confidence intervals from linear mixed models. Estimates of the effect of the individual interventions (pH7 and pH9) and the contrast between them (pH9 vs. pH7) are depicted separately. Q-values generated *ad modum* Benjamini-Hochberg are shown in case of nominal significance.

FIGURE S2. BASELINE COMPOSITION AT PHYLUM, CLASS AND ORDER LEVEL

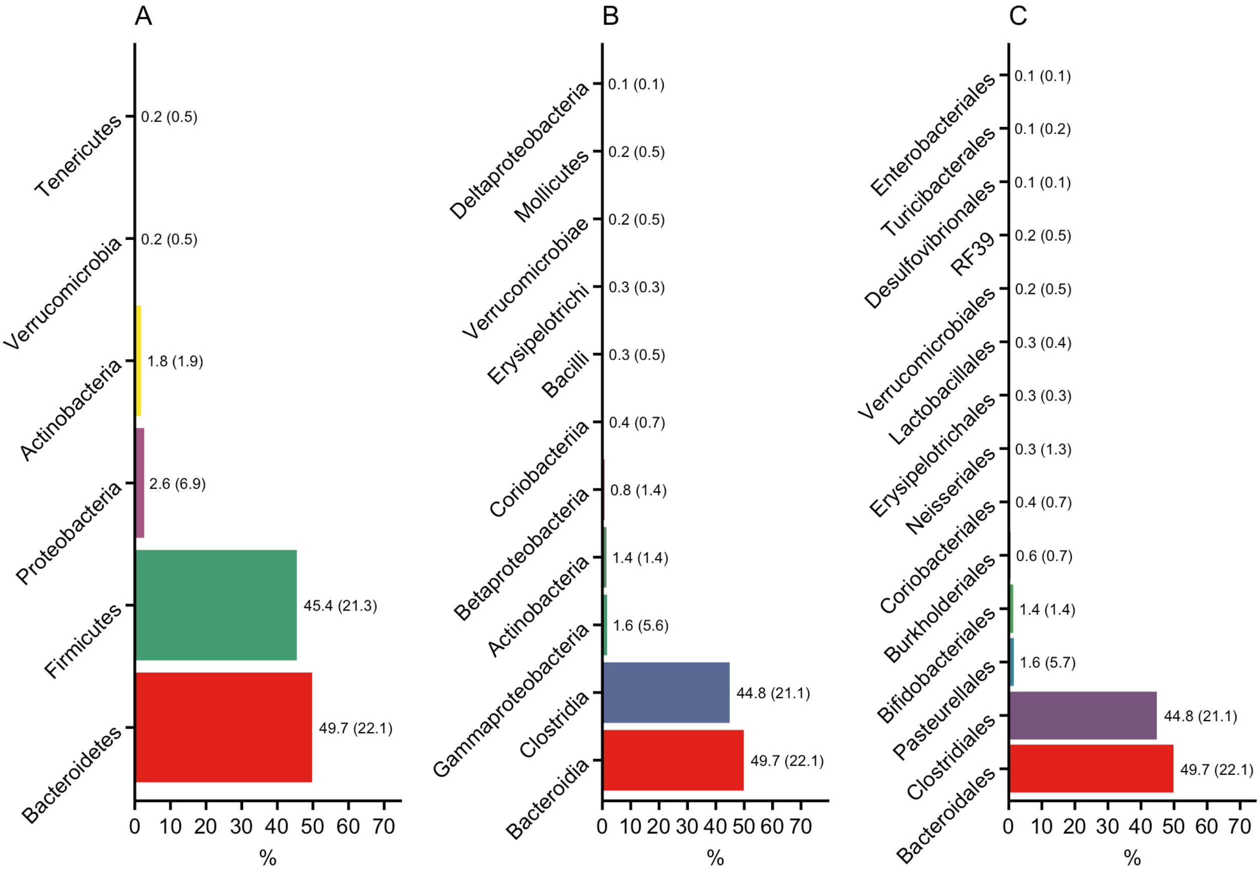

Mean relative abundance (standard error) at phylum [A], class [B] and order [C] level of the 201 core OTUs in baseline samples from all participants.

FIGURE S3. BASELINE COMPOSITION AT FAMILY AND GENUS LEVEL

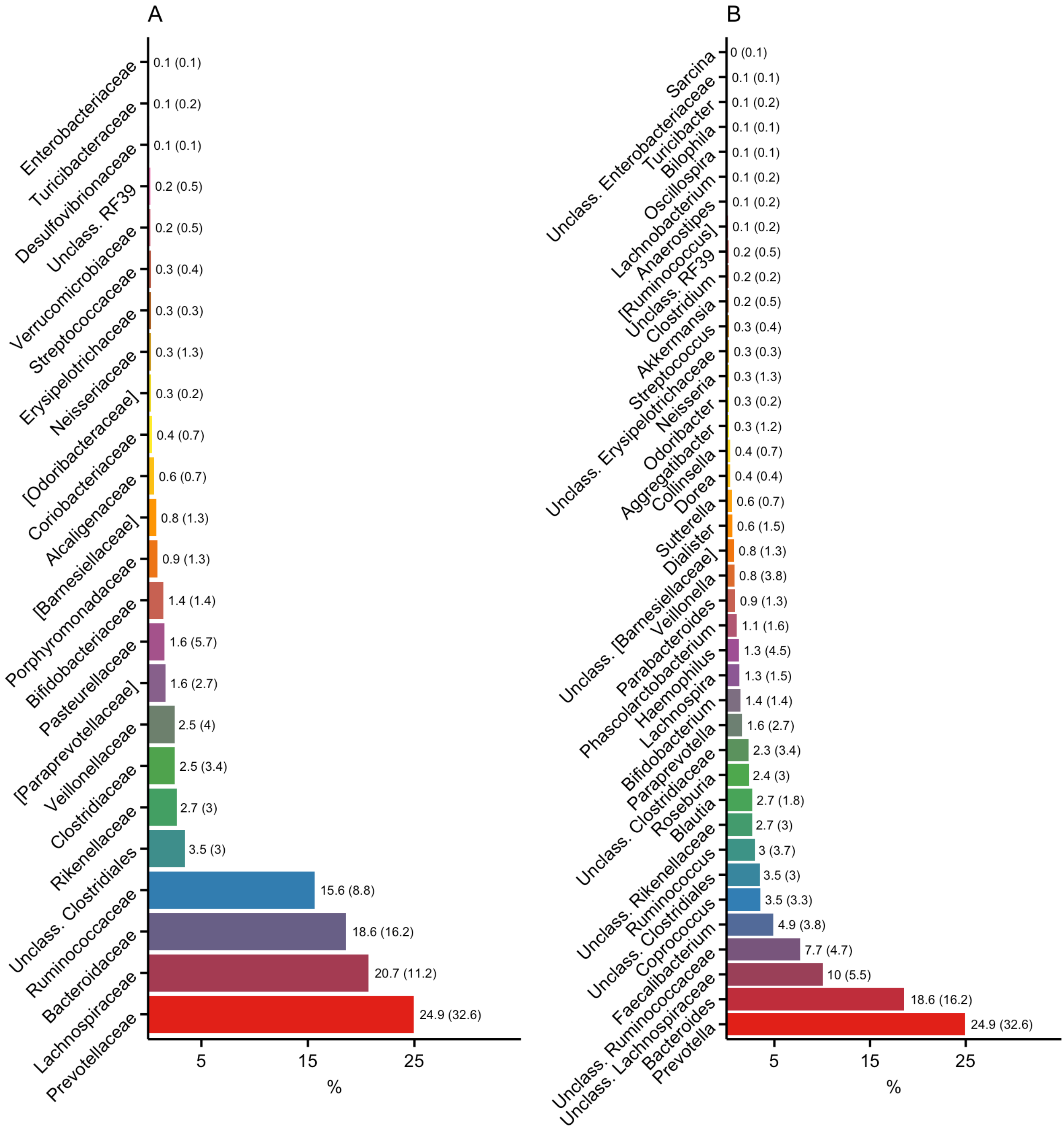

Mean relative abundance (SEM) at family [A] and genus [B] level of the 201 core OTUs in baseline samples from all participants.

FIGURE S4. FOREST PLOT OF INTERVENTION EFFECT IN EACH PERIOD

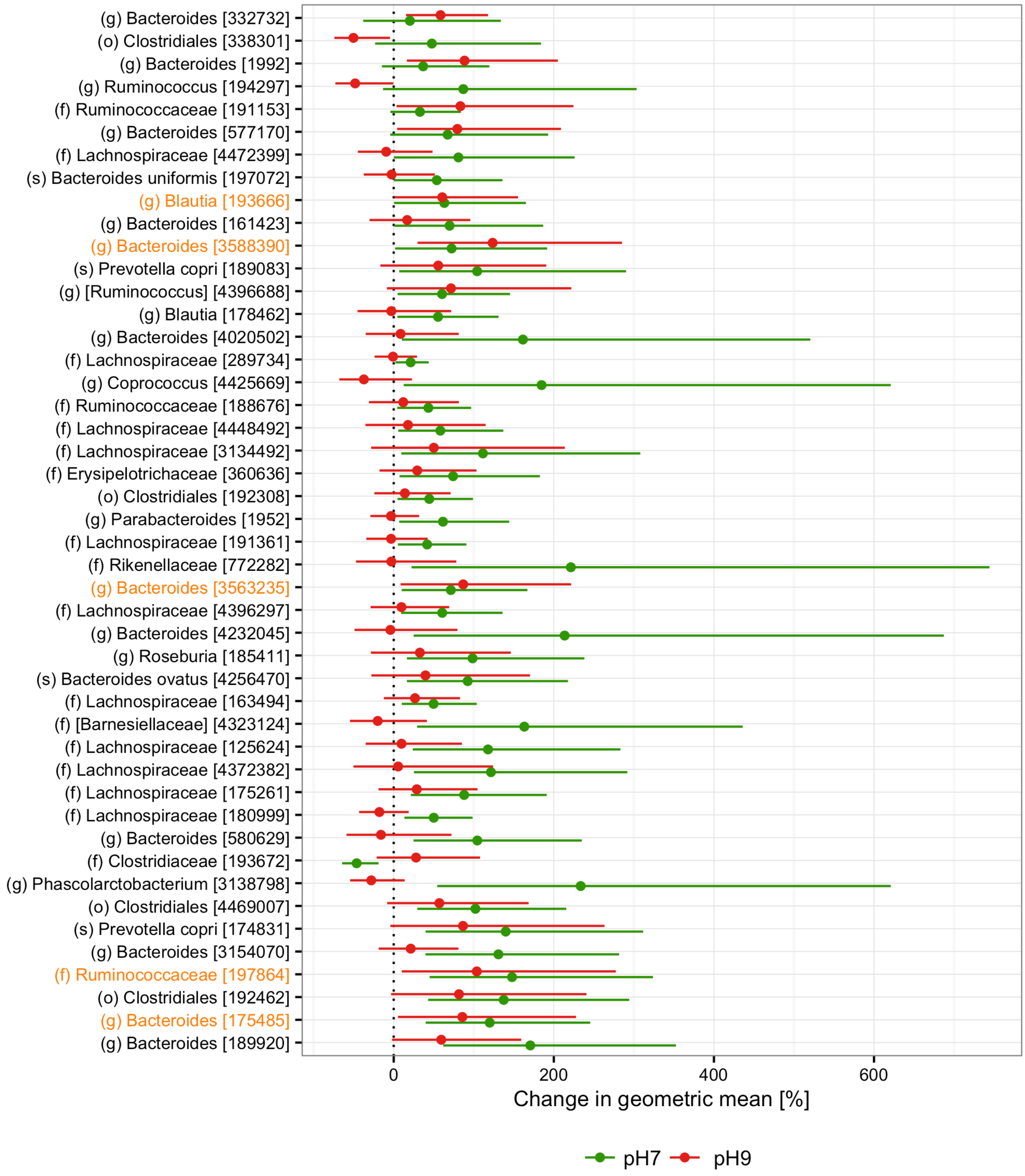

Forest plot of change in relative abundance (% change in geometric mean and 95% confidence intervals) during the individual interventions (pH7 and pH9). Taxonomy of OTUs [Greengenes ID] is given at the lowest classified rank. Only OTUs for which change in relative abundance was nominally significant (P≤0.05) during either intervention are depicted. OTUs for which change was nominally significant during both interventions, with similar direction of effect, are highlighted. o, order. f, family. g, genus. s, species.

FIGURE S5. FOREST PLOT OF COMMON INTERVENTION EFFECTS

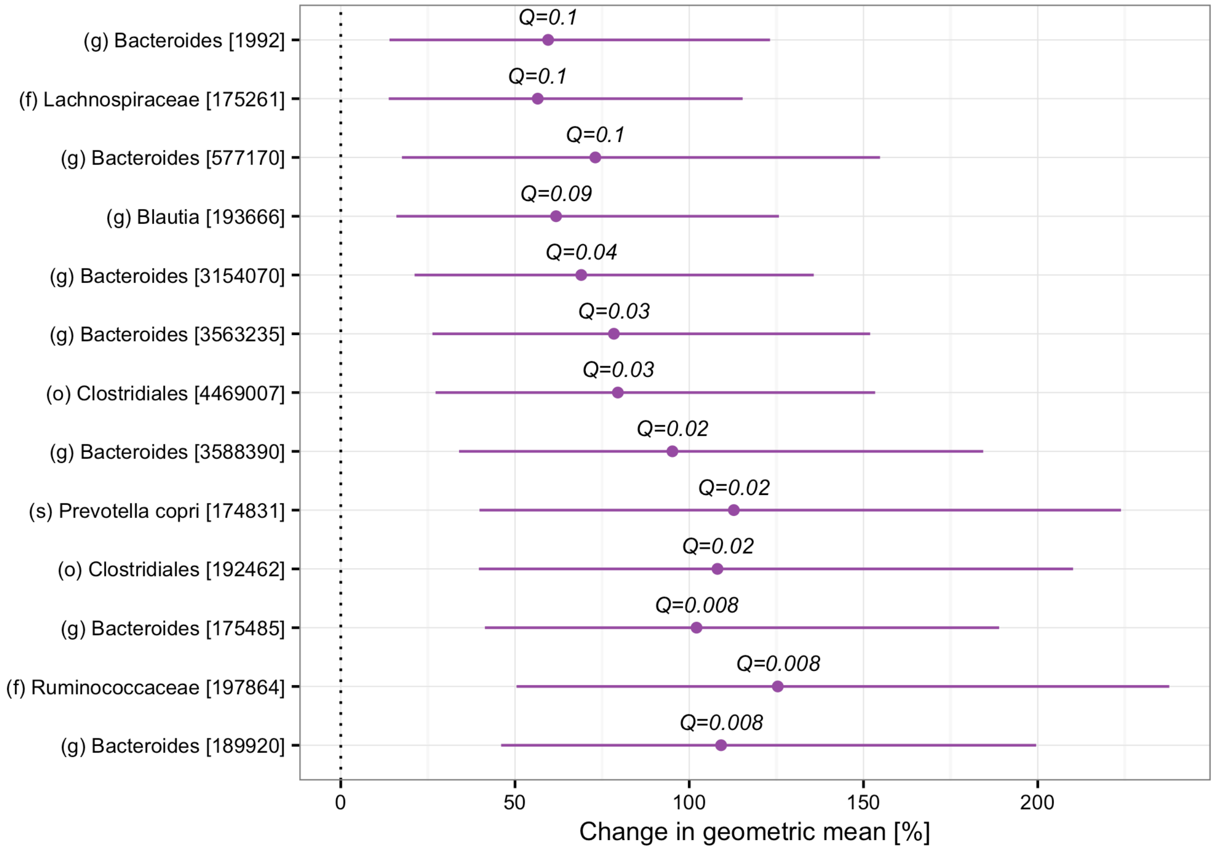

Forest plot of change in relative abundance (% change in geometric mean and 95% confidence intervals) when considering paired samples from the two interventions as technical replicates. Taxonomy of OTUs [Greengenes ID] is given at the lowest classified rank. Only OTUs for which change in relative abundance was nominally significant ($P \leq 0.05$) during either intervention are depicted. Q-values generated *ad modum* Benjamini-Hochberg are shown. o, order. f, family. g, genus. s, species.

FIGURE S6. GLUCOSE AND INSULIN RESPONSE CURVES DURING ORAL GLUCOSE TOLERANCE TEST

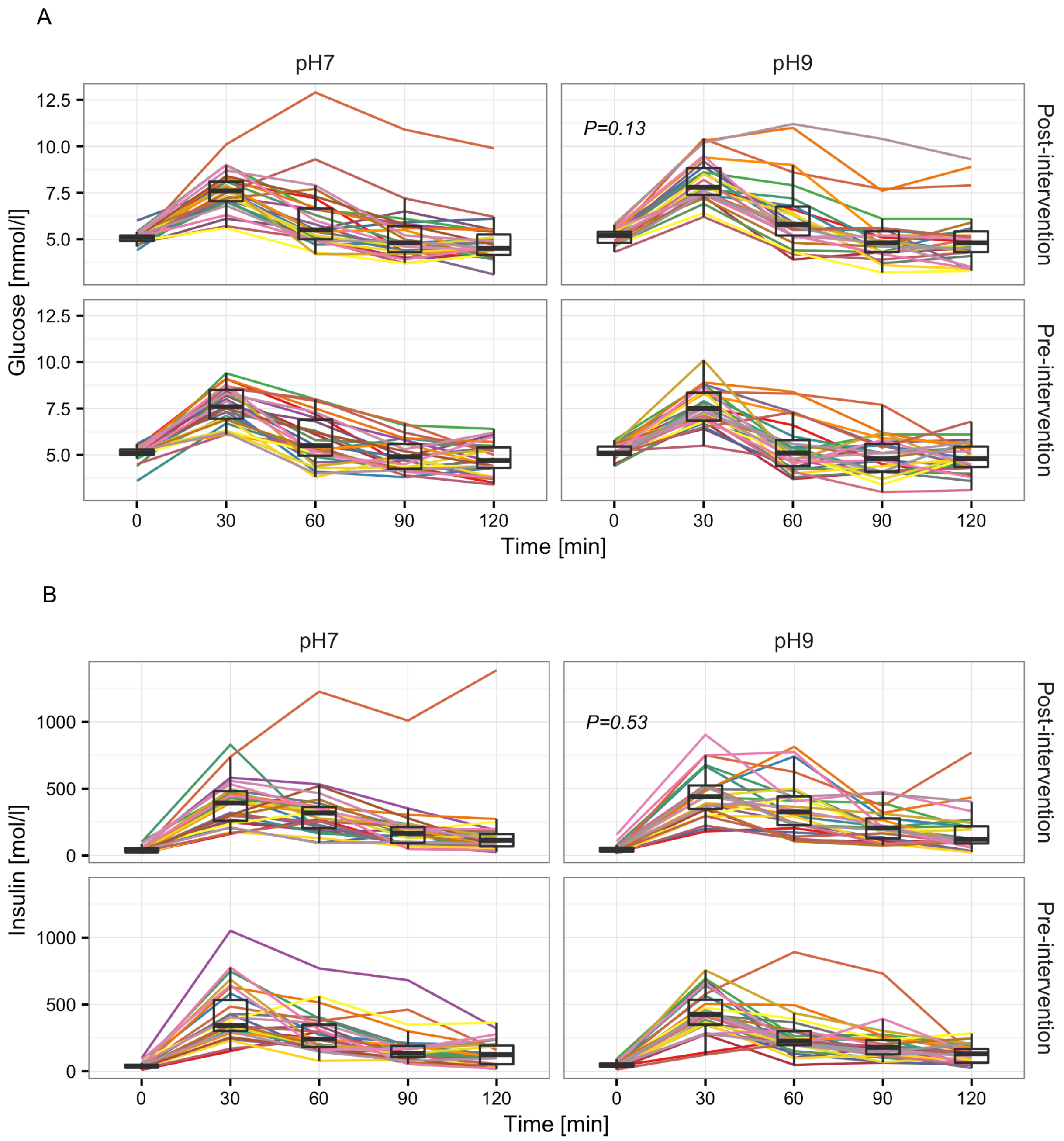

Individual glucose [A] and insulin [B] response curves during an oral glucose tolerance test faceted by type of intervention and sampling time. Each participant is represented by a unique line. Boxplots summarize measurements across participants at individual time-points. Treatment effect (alkaline relative to neutral water) was estimated using linear mixed modelling considering all sampled time-points (global test), and the respective P-values are indicated.

FIGURE S7. GASTRO-INTESTINAL SYMPTOMS

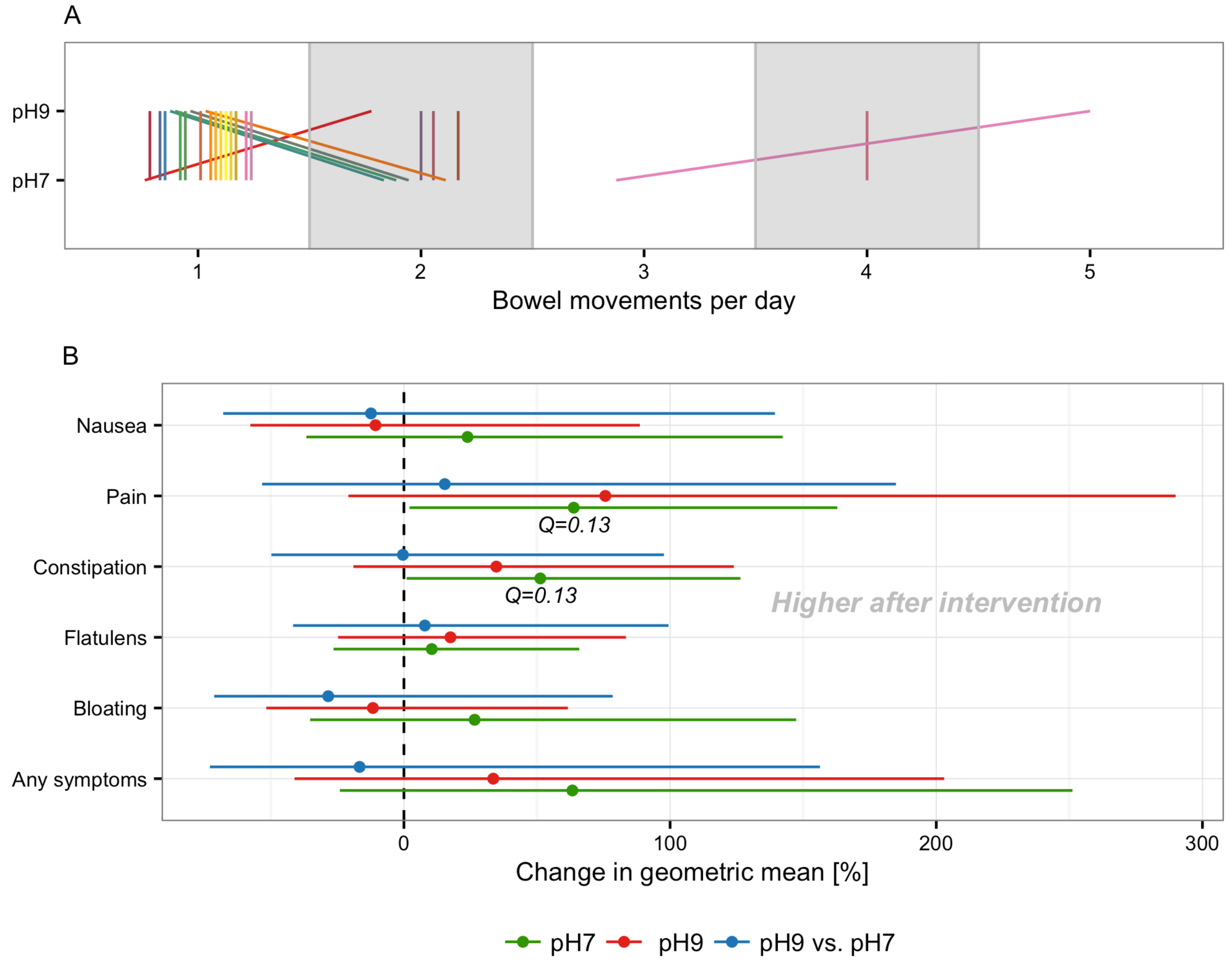

[A] Frequency of bowel movements was not significantly different (Wilcoxon signed-rank test, P=0.82) at post-intervention visits (completers only). Each participant is represented by a unique line. [B] Forest plot of effect estimates (% change in geometric means) and 95% confidence intervals from linear mixed models. Estimates of the individual interventions (pH7 and pH9) and the contrast between them (pH9 vs. pH7) are depicted separately. Q-values generated *ad modum* Benjamini-Hochberg are shown in case of nominal significance.

Table S1. Indices of insulin resistance and beta-cell function

Index	Description	Calculation
BIGTT AIR_{30}	Estimate of acute insulin response (0-30min) [1]	$\exp[8.20 + (0.00178 \times I_0) + (0.00168 \times I_{30}) - (0.000383 \times I_{120}) + (0.314 \times G_0) - (0.109 \times G_{30}) + (0.0781 \times G_{120}) + (0.180 \times sex) + (0.032 \times BMI)]$
CIR	Corrected Insulin response; glucose stimulated insulin secretion	$(100 \times I_{30}) / (G_{30} \times (G_{30} - 3.89))$
DI	Oral Disposition Index; β-cell function adjusted for insulin sensitivity	$IGI/ISI_{Matsuda}$
HOMA2-B	Homeostatic Model Assessment of β-cell function (fasting β-cell function)[2]	Calculator available at: https://www.dtu.ox.ac.uk/homacalculator/
HOMA2-IR	Homeostatic Model Assessment of Insulin Resistance (fasting β-cell function)[2]	Calculator available at: https://www.dtu.ox.ac.uk/homacalculator/
IGI	Insulinogenic Index; glucose stimulated insulin secretion	$(I_{30} - I_0)/ (G_{30} - G_0)$
$ISI_{Matsuda}$	Insulin sensitivity index; approximation of whole-body insulin sensitivity [3]	$10000/sqrt(G_0 \times I_0 \times G_{mean} \times I_{mean})$
MCR	Metabolic Clearance Rate [4]	$18.8 - 0.271 \times BMI - 0.0052 \times I_{120} - 0.27 \times G_{90}$
OGIS	Oral Glucose Insulin Index; insulin sensitivity [5]	Calculator available at: http://webmet.pd.cnr.it/ogis/
PH1	First-phase insulin release [4]	$1.283+1.829 \cdot I_{30}-138.7 \cdot G_{30}+3.772 \cdot I_0$
PH2	Second-phase insulin release [4]	$287+0.4164 \cdot I_{30}-26.07 \cdot G_{30}+0.9226 \cdot I_0$
QUICKI	Quantitative insulin sensitivity check index [6]	$1/(\log(I_0) + \log(G_0))$
$ISI_{Stumvoll}$	Insulin sensitivity index [7]	$1194 + 4.724 \times I_0 - 117.0 \times G_{60} + 1.414 \times I_{60}$

TABLE S2. BETWEEN-PERIOD INTERVENTION EFFECT AT GENUS LEVEL

OTU	Level	Taxonomy	Estimate	97.5 CI	2.5 CI	P	Q
3138798	Genus	Phascolarctobacterium	-76.4	-41.6	-90.5	0.002	0.07
4396235	Genus	Neisseria	-60.4	-10.4	-82.5	0.03	0.52
4472721	Genus	Odoribacter	-42.7	2.3	-67.9	0.06	0.79
193672	Family	Clostridiaceae	54.6	191.2	-17.9	0.18	0.98
1952	Genus	Parabacteroides	-28.2	19.2	-56.8	0.2	0.98
197004	Family	Lachnospiraceae	-13.5	9.2	-31.5	0.22	0.98
4453609	Family	Rikenellaceae	54	209.9	-23.5	0.23	0.98
368490	Genus	Turicibacter	75	406.1	-39.5	0.3	0.98
3943186	Genus	Lachnobacterium	65.8	428	-47.9	0.39	0.98
360636	Family	Erysipelotrichaceae	-23.6	44.5	-59.6	0.41	0.98
195937	Genus	Blautia	-11.4	19.1	-34.1	0.42	0.98
4453501	Genus	Veillonella	-23.8	48.8	-61	0.43	0.98
4437359	Genus	Oscillospira	28.2	163.8	-37.7	0.5	0.98
4306262	Genus	Akkermansia	-26.3	80.2	-69.9	0.5	0.98
163243	Genus	Ruminococcus	19.4	107	-31.2	0.53	0.98
4363066	Genus	Aggregatibacter	50.2	438.5	-58.1	0.53	0.98
175844	Family	[Barnesiellaceae]	-24.4	88.7	-69.7	0.55	0.98
176980	Genus	Dorea	17	97.6	-30.7	0.56	0.98
4425214	Genus	Streptococcus	-25.7	124.2	-75.4	0.6	0.98
359872	Genus	Bilophila	-12.6	70	-55.1	0.69	0.98
4449236	Genus	Sutterella	-14.7	99.4	-63.5	0.71	0.98
4428714	Order	RF39	-21.3	190.8	-78.7	0.72	0.98
199145	Genus	Faecalibacterium	-6.1	40.5	-37.3	0.76	0.98
4425669	Genus	Coprococcus	-4.8	33	-31.9	0.77	0.98
181432	Genus	Paraprevotella	12.5	149.3	-49.3	0.77	0.98
4436552	Genus	Prevotella	-10.5	94.9	-58.9	0.78	0.98
3600504	Genus	Bacteroides	-7.1	61.5	-46.5	0.79	0.98
4396688	Genus	[Ruminococcus]	7.6	98.8	-41.8	0.82	0.98
181059	Order	Clostridiales	-3.3	30	-28.1	0.82	0.98
782953	Family	Enterobacteriaceae	-12.3	216.8	-75.7	0.84	0.98
4481613	Genus	Collinsella	-5.6	73.7	-48.7	0.85	0.98
403701	Genus	Dialister	-7.3	126.6	-62.1	0.87	0.98
298937	Genus	Sarcina	-14.4	436.9	-86.4	0.87	0.98
3931537	Genus	Clostridium	-4.8	96.8	-53.9	0.89	0.98
3537197	Genus	Anaerostipes	-4.8	101.7	-55	0.9	0.98
177515	Genus	Roseburia	2.2	75.7	-40.6	0.94	0.98
4347159	Genus	Bifidobacterium	3.3	143.1	-56.1	0.94	0.98
4477696	Genus	Haemophilus	-3.4	164.1	-64.7	0.95	0.98
176269	Genus	Lachnospira	-1.6	86	-48	0.96	0.98
265871	Family	Ruminococcaceae	-0.1	37.8	-27.5	1	1

Estimates are change in geometric means and corresponding 95% confidence intervals from linear mixed models contrasting pH9 to pH7. Q values are calculated *ad modum* Bejamini-Hochberg. The taxonomic level to which an OTU is annotated and the corresponding name is presented.

TABLE S3. WITHIN-PERIOD INTERVENTION EFFECT AT GENUS LEVEL

OTU	Level	Taxonomy	Intervention	Estimate	97.5 CI	2.5 CI	P	Q
197004	Family	Lachnospiraceae	pH7	21.4	37.7	7.0	0.003	0.06
3138798	Genus	Phascolarctobacterium	pH7	214.7	570.8	47.6	0.003	0.06
360636	Family	Erysipelotrichaceae	pH7	68.7	167.5	6.4	0.03	0.35
1952	Genus	Parabacteroides	pH7	40.6	104.8	-3.4	0.08	0.48
195937	Genus	Blautia	pH7	16.0	37.1	-1.9	0.08	0.48
4396235	Genus	Neisseria	pH7	39.6	99.9	-2.6	0.07	0.48
4396688	Genus	[Ruminococcus]	pH7	35.5	88.6	-2.7	0.07	0.48
163243	Genus	Ruminococcus	pH7	22.7	74.1	-13.5	0.25	0.54
175844	Family	[Barnesiellaceae]	pH7	57.8	205.5	-18.5	0.18	0.54
176980	Genus	Dorea	pH7	19.4	62.4	-12.2	0.26	0.54
177515	Genus	Roseburia	pH7	27.5	71.4	-5.2	0.11	0.54
193672	Family	Clostridiaceae	pH7	-23.6	11.9	-47.8	0.17	0.54
199145	Genus	Faecalibacterium	pH7	14.0	40.0	-7.2	0.21	0.54
3600504	Genus	Bacteroides	pH7	23.9	71.5	-10.5	0.20	0.54
3943186	Genus	Lachnobacterium	pH7	-35.8	27.5	-67.6	0.21	0.54
4363066	Genus	Aggregatibacter	pH7	-40.3	39.4	-74.5	0.23	0.54
4449236	Genus	Sutterella	pH7	42.2	145.8	-17.7	0.21	0.54
4453609	Family	Rikenellaceae	pH7	-22.1	19.0	-49.0	0.25	0.54
782953	Family	Enterobacteriaceae	pH7	69.9	325.6	-32.2	0.26	0.54
4453501	Genus	Veillonella	pH7	14.3	53.4	-14.8	0.37	0.74
4306262	Genus	Akkermansia	pH7	39.3	203.5	-36.1	0.40	0.77
4472721	Genus	Odoribacter	pH7	18.7	81.5	-22.4	0.43	0.78
181059	Order	Clostridiales	pH7	7.8	32.5	-12.3	0.47	0.79
298937	Genus	Sarcina	pH7	21.8	132.9	-36.3	0.55	0.79
3537197	Genus	Anaerostipes	pH7	14.7	92.5	-31.6	0.60	0.79
359872	Genus	Bilophila	pH7	-12.2	45.6	-47.0	0.61	0.79
368490	Genus	Turicibacter	pH7	-19.6	81.4	-64.3	0.60	0.79
403701	Genus	Dialister	pH7	23.5	115.0	-29.1	0.46	0.79
4425669	Genus	Coprococcus	pH7	4.9	26.2	-12.8	0.61	0.79
4428714	Order	RF39	pH7	-26.8	136.4	-77.3	0.60	0.79
4436552	Genus	Prevotella	pH7	13.5	72.2	-25.3	0.55	0.79
181432	Genus	Paraprevotella	pH7	-10.7	64.2	-51.5	0.72	0.87
3931537	Genus	Clostridium	pH7	-9.9	51.9	-46.6	0.69	0.87
4425214	Genus	Streptococcus	pH7	13.3	146.7	-48.0	0.75	0.89
4477696	Genus	Haemophilus	pH7	-8.8	83.2	-54.6	0.80	0.91
265871	Family	Ruminococcaceae	pH7	-1.9	16.6	-17.5	0.83	0.92
176269	Genus	Lachnospira	pH7	4.1	61.0	-32.7	0.86	0.93

TABLE S3 (CONTINUED)

OTU	Level	Taxonomy	Intervention	Estimate	97.5 CI	2.5 CI	P	Q
4437359	Genus	Oscillospira	pH7	3.1	69.4	-37.3	0.91	0.95
4347159	Genus	Bifidobacterium	pH7	-0.8	71.3	-42.5	0.98	0.99
4481613	Genus	Collinsella	pH7	-0.2	54.6	-35.6	0.99	0.99
4428714	Order	RF39	pH9	-38.4	3.9	-63.4	0.07	0.87
4472721	Genus	Odoribacter	pH9	-29.7	3.1	-52.1	0.07	0.87
4396235	Genus	Neisseria	pH9	-47.8	8.4	-74.8	0.08	0.87
163243	Genus	Ruminococcus	pH9	44.6	120.5	-5.2	0.09	0.87
3138798	Genus	Phascolarctobacterium	pH9	-29.4	11.0	-55.1	0.13	0.94
176980	Genus	Dorea	pH9	38.9	118.2	-11.5	0.15	0.94
4396688	Genus	[Ruminococcus]	pH9	43.0	143.8	-16.1	0.19	0.94
359872	Genus	Bilophila	pH9	-24.2	15.2	-50.1	0.19	0.94
360636	Family	Erysipelotrichaceae	pH9	25.0	92.3	-18.7	0.31	0.94
403701	Genus	Dialister	pH9	36.5	156.5	-27.4	0.33	0.94
368490	Genus	Turicibacter	pH9	43.4	208.2	-33.2	0.36	0.94
177515	Genus	Roseburia	pH9	23.7	95.6	-21.8	0.36	0.94
193672	Family	Clostridiaceae	pH9	22.9	101.7	-25.1	0.41	0.94
4437359	Genus	Oscillospira	pH9	26.5	124.9	-28.9	0.42	0.94
4453609	Family	Rikenellaceae	pH9	26.4	125.6	-29.2	0.43	0.94
4306262	Genus	Akkermansia	pH9	18.2	79.3	-22.0	0.43	0.94
782953	Family	Enterobacteriaceae	pH9	37.0	219.8	-41.3	0.47	0.94
3600504	Genus	Bacteroides	pH9	17.9	87.1	-25.8	0.49	0.94
3537197	Genus	Anaerostipes	pH9	19.2	108.2	-31.7	0.54	0.94
3931537	Genus	Clostridium	pH9	-13.5	41.0	-46.9	0.56	0.94
4453501	Genus	Veillonella	pH9	-16.0	53.0	-53.9	0.57	0.94
4449236	Genus	Sutterella	pH9	22.9	153.3	-40.3	0.58	0.94
175844	Family	[Barnesiellaceae]	pH9	20.0	129.7	-37.3	0.58	0.94
4425214	Genus	Streptococcus	pH9	-19.7	75.0	-63.1	0.58	0.94
4477696	Genus	Haemophilus	pH9	-17.9	71.4	-60.6	0.60	0.94
298937	Genus	Sarcina	pH9	42.5	524.0	-67.5	0.64	0.94
4363066	Genus	Aggregatibacter	pH9	-17.0	117.0	-68.3	0.70	0.94
3943186	Genus	Lachnobacterium	pH9	16.7	188.6	-52.8	0.74	0.94
181432	Genus	Paraprevotella	pH9	8.6	81.8	-35.2	0.75	0.94
181059	Order	Clostridiales	pH9	3.3	29.8	-17.8	0.78	0.94
199145	Genus	Faecalibacterium	pH9	4.5	48.8	-26.6	0.81	0.94
197004	Family	Lachnospiraceae	pH9	2.5	26.7	-17.0	0.82	0.94
4481613	Genus	Collinsella	pH9	-4.7	44.3	-37.1	0.82	0.94
4425669	Genus	Coprococcus	pH9	-3.1	29.3	-27.4	0.83	0.94

TABLE S3 (CONTINUED)

OTU	Level	Taxonomy	Intervention	Estimate	97.5 CI	2.5 CI	P	Q
4347159	Genus	Bifidobacterium	pH9	6.6	108.7	-45.6	0.85	0.94
265871	Family	Ruminococcaceae	pH9	-1.9	29.1	-25.5	0.89	0.94
176269	Genus	Lachnospira	pH9	3.0	63.9	-35.3	0.90	0.94
195937	Genus	Blautia	pH9	1.4	29.6	-20.7	0.91	0.94
4436552	Genus	Prevotella	pH9	3.5	101.1	-46.8	0.92	0.94
1952	Genus	Parabacteroides	pH9	-0.6	36.0	-27.3	0.97	0.97

Estimates are percentual change in geometric means and corresponding 95% confidence intervals from linear mixed models of intervention effect within each period. Q Values are calculated ad modum Bejamini-Hochberg.
